# Improved Detection of Minimal Residual Disease in AML: Validation of IDH1/2 ddPCR Assays in the Perspective of Treatment with Target Inhibitors

**DOI:** 10.3390/ijms262110397

**Published:** 2025-10-26

**Authors:** Katsiaryna Nikitsenka, Giacomo Danieli, Lucia Tombolan, Barbara Mancini, Davide Facchinelli, Giorgia Scotton, Alberto Tosetto, Omar Perbellini, Daniela Zuccarello, Elisabetta Novella

**Affiliations:** 1 Medical Genetics and Genomic Unit, San Bortolo Hospital, 36100 Vicenza, Italy; 2 Hematology Unit, San Bortolo Hospital, 36100 Vicenza, Italy; 3 Public Health and Prevention Department, San Bortolo Hospital, 36100 Vicenza, Italy

**Keywords:** *IDH* gene, ddPCR, *IDH1* R132H, *IDH1* R132C, *IDH2* R140Q, *IDH2* R172K, LOB, LOD, AML molecular markers, MRD

## Abstract

Mutations in *IDH1/2* are frequent in Acute Myeloid Leukemia (AML), defining a molecularly distinct subgroup with therapeutic implications due to the availability of specific inhibitors. Accurate monitoring of treatment response is crucial and Droplet Digital PCR (ddPCR) offers a sensitive approach for quantifying mutational burden in *IDH*-mutated AML. This study aimed to optimize and validate ddPCR assays specific for *IDH1 R*132 and *IDH2 R*172/*R*140 mutations for future use in Minimal Residual Disease (MRD) monitoring. Four ddPCR assays were set to evaluate the trend of *IDH1/2* mutations in 191 diagnostic and follow-up samples. Each validation procedure included determining the limit of blank (LOB) and limit of detection (LOD) using titration series. Moreover, in AML harboring both *IDH* and *NPM1* mutations, we performed generalized estimating equations (GEE) to assess the association between *IDH* fractional abundance and *NPM1* RQ-Ratio across time points. Four *IDH1/2* ddPCR assays were validated, demonstrating high sensitivity with limits of detection of 0.07% for *IDH1* R132H, 0.1% for *IDH*2 R140Q and R172K, and 0.2% for *IDH1* R132C. The method also exhibited excellent intra-run reproducibility, providing consistent results for patient follow-up. Comparison of *IDH* and *NPM*1 trends during follow-up revealed a statistically significant positive correlation, both in raw (β = 0.079, *p* = 0.001) and ranked data (β = 0.99, *p* = 0.004), suggesting a co-dynamic pattern potentially useful for surrogate monitoring. While our study cannot yet define the clinical role of *IDH* mutation assessment by ddPCR due to the lack of comparative follow-up studies, it establishes a solid methodological foundation for standardizing minimal residual disease evaluation via ddPCR, paving the way for future prospective validation.

## 1. Introduction

Acute Myeloid Leukaemia is a highly heterogeneous hematological disease with acute onset, poor prognosis, chemotherapy resistance and frequent relapses. The precise molecular characterization of AML is crucial for defining risk stratification, selecting the most appropriate therapeutic strategy and monitoring the minimal residual disease [1,2].

Mutations in Isocitrate Dehydrogenase (IDH) enzymes occur in approximately 15–20% of all AML cases [3]. In cases harboring *IDH* mutations, the metabolic pathways reliant on α-KG are disrupted, resulting in epigenetic dysregulation, which includes abnormal histone and DNA methylation, chromatin remodeling and blockage of cellular differentiation, all contributing to transformative effects [3,4].

The prognostic significance of mutated IDH in patients with AML is still debated and its impact appears to be contingent on clinical context, co-occurring mutational profile (e.g., *NPM1, FLT3-ITD*, *DNMT3A*, complex karyotype, etc.) and hot spot location [4,5,6]. Recent studies showed that *IDH2* R172K mutations may confer an extremely poor prognosis. Consequently, some authors suggest classifying patients with this unique molecular profile into a distinct AML subgroup [7,8]. Although co-mutations can influence outcomes in AML patients with *IDH1* R132 and *IDH2* R140Q, these *IDH* mutations frequently appear alongside *NPM1* or *DNMT3A*, both established indicators of a favorable prognosis. Significantly, the 2024 ELN recommendations now categorize all patients with *IDH1*-mutated AML as having a favorable prognosis within the context of AZA/IVO therapy [9,10,11].

The clinical utility of *IDH* gene mutations as pharmacological targets in both relapsed/refractory AML and newly diagnosed patients has been extensively investigated in recent years. Clinical trials evaluating specific IDH inhibitors, such as Ivosidenib (*IDH1*) and Enasidenib (*IDH*2), have demonstrated significant efficacy [1]. Since May 2023, the European Medicines Agency (EMA) has approved Ivosidenib for the treatment of newly diagnosed adult AML patients harboring *IDH1* R132 mutations but deemed medically unfit for IC, both in combination with azacitidine or alone in patients >75 years old or with comorbidities. At the moment, Enasidenib, however, remains under investigation in European clinical trials [12].

Given therapeutic developments with *IDH1* inhibitors, the accurate quantification of these mutations may be determinant in management of AML patients for assessing their response to new treatment [13]. Digital droplet PCR has demonstrated its high sensitivity for absolute quantification of very low mutated copies in abundant wild type background (up to 10^−4^ log), and can be potentially used in laboratory routine alongside flow cytometry, thus satisfying the clinical need to produce fast results and compete with Next Generation Sequencing (NGS) which is the standard for *IDH1/2* mutation detecting, but still remains expensive and time consuming [14,15].

Our study focused on optimizing and validating four commercial ddPCR assays for the *IDH1* R132H/C, *IDH*2 R140Q, and *IDH*2 R172K mutations. The goal was to implement them for clinical minimal residual disease (MRD) monitoring. After establishing the assays’ performance characteristics—including limit of blank, limit of detection, accuracy, and reproducibility—we applied them to a specific patient cohort. This cohort included patients with isolated IDH mutations and a subgroup with co-expressing *NPM*1, a known prognostic MRD marker in AML. Our objective was to observe trends for both targets and determine if *IDH* could serve as a reliable surrogate marker for treatment efficacy in patients with a sole *IDH* mutation.

## 2. Results and Discussion

### 2.1. The IDH1/IDH2 Mutation Distribution

We identified *IDH1* or *IDH*2 mutations in 45 out of 215 (20.9%) AML patients studied. This mutational distribution confirms data observed in other publications [16]. Patients’ molecular profiles are reported in Table 1.

### 2.2. Analytical Performance of ddPCR Assays

Following optimization of ddPCR amplification conditions, the average background signal was calculated using 29 blank samples (*IDH1* R132H), 40 blank samples (*IDH1* R132C), and 30 wildtype samples for both *IDH2* R140Q and R172K variants. Data from blank samples define the probability of false-positive background presence, which is expected in all the unknown samples to be analysed. The LOB, in terms of fractional abundance, was determined to be 0.05% for *IDH1* R132H and 0.02% for *IDH*2 R140Q, *IDH1* R132C, and *IDH*2 R172K mutations (Table 2).

The LOD was calculated through a serial dilution of 7 low-level (LL) mutation-positive samples into wild-type samples to achieve a variant allele frequency of 0.05%, simulating varying allele burdens. For *IDH1* codon 132 (R132C/H) and *IDH*2 codon 140 the range between dilution points were chosen to be with small increments in concentration: 30, 15, 10, 8, 4, 2 and 1 copies/reaction and a minimum of eight replicates was performed at each concentration to assess precision across all dilution points. For *IDH*2 codon 172 (R172K) dilution points were chosen as 20, 10, 5, 2.5, 1.5 and 1 copies/reaction and a minimum of five replicates was performed at each concentration. Results are presented in Table 3. Minimal variation was observed between replicates, which indicates reliability of four assays.

### 2.3. Linearity Assessment via Log-Log Regression

**Figure 1:**
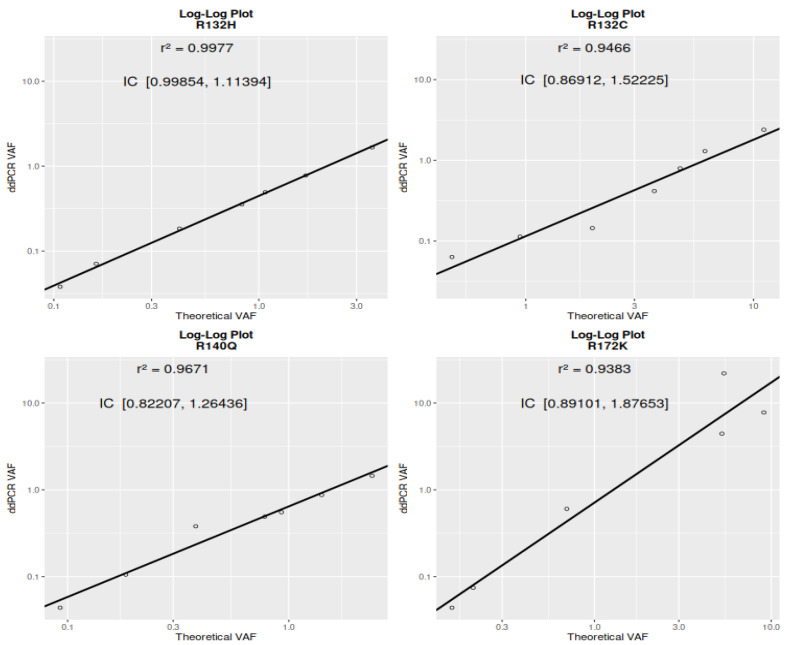
Four graphs represent the linearity of droplet ddPCR in quantifying variant allelic frequency (VAF). The x-axis represents the theoretical VAF, calculated by multiplying the undiluted DNA sample’s *IDH1* or *IDH*2 mutant VAF by the corresponding dilution ratio. Each data point on the graphs represents the mean VAF obtained from eight replicates using ddPCR. The coefficient of determination (r^2^) is displayed on each graph to indicate the model fit.

A log-log regression analysis was performed to assess the relationship between theoretical and measured VAFs for the mutations R132H, R132C, R140Q, and R172K. Figure 1 shows that the strongest linearity was observed for R132H, with a regression slope (β) of 1.06–1.11 (95% CI: [0.999, 1.114]) and an R^2^ of 0.998, indicating excellent quantitative agreement and proportionality across the dilution series. R132C and R140Q also showed strong linear relationships, with β confidence intervals of [0.869, 1.522] and [0.822, 1.264], respectively, and R^2^ values of 0.947 and 0.967. Although their intervals include 1, suggesting no significant deviation from proportionality, they reflect high accuracy and reliable quantification. R172K showed a higher slope (β = 1.38 [0.891, 1.877]) and a good fit (R^2^ = 0.938), but with a wider confidence interval compared to the other mutations, indicating slightly greater variability. The variability from linearity suggests that pipetting error may be a contributing factor. Overall, the results confirm strong log-linear behavior for all targets, particularly R132H, and support the reliability of ddPCR quantification within the tested VAF range.

### 2.4. IDH and NPM1 Mutation Dynamics

We selected 6 patients who co-expressed both *NPM1* and *IDH1/2* mutations (Table 4), based on the availability of at least six follow-up monitoring samples for each. A total of 68 samples were evaluated. None of the patients in this study received IDH-targeted inhibitors.

The samples with both *NPM1* and *IDH1* values below the detection limit were excluded from correlation analyses. This choice reflects the aim of evaluating whether *IDH*1 fractional abundance reliably mirrors *NPM1* dynamics in samples where at least one marker is detectable, as double-undetectable cases do not contribute information on marker co-variation.

**Figure 2:**
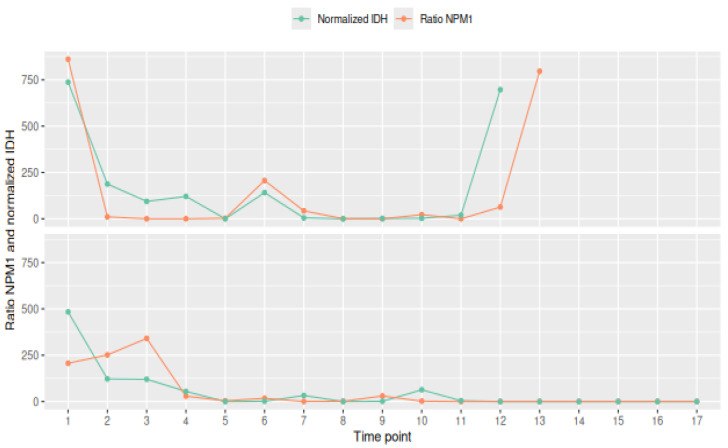
Two examples of representative trends. A normalized *IDH* value (idh_norm) was then computed by scaling the original *IDH* fractional abundance using the ratio between the total *NPM1* and *IDH* values.

The GEE analysis confirmed a statistically significant positive relationship between the two markers, both on raw values (β = 0.04618, *p* < 0.001) and on ranked data (β = 0.6509, *p* < 0.001), suggesting robust monotonic co-dynamics. These findings support the potential for mutual surrogate monitoring in this patient subgroup, although further validation in larger cohorts is warranted (Figure 2).

### 2.5. Discussion

Distinguishing between residual leukemia and benign clonal hematopoiesis is crucial for accurate clinical interpretation and for validating the role of *IDH* in measurable residual disease monitoring. This is particularly important because *IDH1/2* mutations and other common variants like *DNMT3A* can occur in age-related clonal hematopoiesis (CHIP) independently of overt leukemia, especially at low variant allele frequencies without co-occurring leukemic mutations [17]. Longitudinal tracking of VAF dynamics could provide valuable information; stable *IDH* VAFs may suggest CHIP, while rising VAFs could indicate an impending relapse [17,18].

Targeted therapies, such as Ivosidenib for *IDH1*-mutated AML, can exert selective pressure on leukemic clones, potentially altering the *IDH* mutation burden over time and leading to clonal evolution or resistance [19]. This dynamic genetic landscape can affect the reliability of molecular techniques like droplet digital PCR for MRD monitoring. *IDH1* inhibitor (Ivosidenib) induces differentiation of leukemic blasts in AML with *IDH1* mutations. However, not all patients achieve complete molecular clearance of the *IDH1* mutation. Studies indicate that a significant proportion of patients in remission after ivosidenib treatment still have detectable *IDH1*-mutant clones. This implies that differentiated cells can retain the *IDH1* mutation even when patients achieve MRD negativity by flow cytometry or NGS. For instance, in one study, only 39–41% of patients achieving complete remission had *IDH1* mutation clearance, whereas MRD-negative remissions by flow cytometry were observed in a higher percentage (up to 89%) [20,21].

The persistence of *IDH1*-mutant cells complicates MRD monitoring because the mutation’s presence may not always signify active disease. Instead, it could reflect residual differentiated, non-leukemic cells or clonal hematopoiesis. Molecular-based MRD assays might detect these persistent *IDH1*-mutant clones, potentially leading to false positives and difficulties in differentiating true relapse from benign clonal populations. Therefore, while ivosidenib is effective in inducing remissions, the retention of the *IDH1* mutation in differentiated cells creates interpretive challenges in MRD monitoring, highlighting the need to carefully integrate molecular and flow cytometric data in clinical decision-making [22].

However, our work provides a solid methodological basis for standardizing minimal residual disease assessment using ddPCR. This is in line with the activity of the European MRD consortium (http://www.euromrd.org), which is actively working to standardize this technique with a view to its integration into routine clinical practice.

## 3. Materials and Methods

### 3.1. AML Patients’ Samples

This study enrolled 215 consecutive newly diagnosed AML patients, according to ELN guidelines [1], diagnosed at the Hematology Unit, San Bortolo Hospital, Vicenza, between May 2019 and October 2024. To explore a possible role of ddPCR assays for monitoring of MRD, we studied 15 patients at diagnosis and during follow-up. These patients were selected based on the availability of samples for a minimum period of six months from diagnosis, treatment and consolidation or subjected to allogeneic haematopoietic stem cell transplantation (alloHSTC). No patients were treated with IDH inhibitors; thus, patients were collected prior to EMA inhibitor approval.

### 3.2. IDH Mutation Analysis

Genomic DNA was extracted from peripheral blood buffy coat and/or bone marrow samples collected in EDTA, using the Promega Maxwell CSC Blood DNA purification kit and the automated Promega Maxwell-16 CSC system (Madison, WI, USA), according to manufacturer’s instructions. All samples showed good absorbance ratios (A260/230 and A230/260).

Prior to July 2022 (91 patients), *IDH1/2* mutations at diagnosis were detected using direct Sanger Sequencing. Sequences were analyzed with an Applied Biosystem 3130 Genetic Analyzer (Waltham, MA, USA) and the BigDye Terminator Cycle Sequencing Kit v1.1 (Thermo Fisher Scientific, Foster city, CI, USA). DNA amplification of *IDH*1 and *IDH*2 exon 4 was performed as previously described.

From July 2022 onwards (124 patients), molecular determination was performed using the EasyPGX-ready *IDH*1/2 IVD kit (Diatech Pharmacogenetics, Ancona, Italy). Reactions followed manufacturer’s specifications and were analyzed with AriaDx Electronic Tracking qPCR Software 2.1 (Santa Clara, CA, USA, Quantitative PCR Data, AriaDx Electronic Tracking qPCR Software|Agilent)

### 3.3. Reaction Setup of ddPCR Assays

Four different ddPCR-specific assays (*IDH1* R132H, *IDH1* R132C, *IDH1* R140Q, and *IDH*2 R172K) developed by Bio-Rad Laboratories (Pleasanton, CA, USA) were used for the quantitative determination of *IDH1/2* mutations in all positive samples collected at diagnosis (n = 45) and in 160 samples collected during follow-up. As these assays were not wet-lab validated by Bio-Rad, they were validated prior to use in our laboratory.

To minimize the “rain” effect, we optimized the hybridization temperature and number of PCR cycles to ensure a better separation between positive and negative droplet populations. We also performed a two-part analysis to determine the lowest concentration of mutated copies: first, the false positive rate was assessed using only wild-type genomic DNA samples, and then mutation titration series were analyzed to define the assay’s sensitivity. Preliminary optimization of the assays was also performed.

Custom primers and probes were specifically designed by Bio-Rad with a combination of either FAM and HEX fluorophores, targeting the mutant and wild-type alleles, respectively. For every reaction, we prepared at least one negative control, containing a DNA concentration similar to that of the unknown samples, and a positive control prepared by diluting 10 ng of mutant DNA in a background of 200 ng of wild-type DNA.

**Figure 3:**
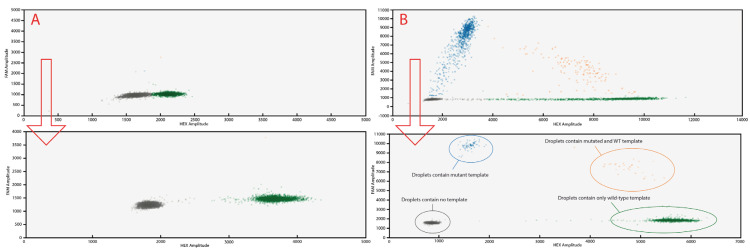
Two-dimensional scatter plot demonstrating changes in cluster separation before and after reaction setup. The red arrow indicates the improvement in performance observed after optimizing the reaction conditions. (**A**)—reaction with the DNA contained only the wild-type sample. (**B**)—reaction with a sample containing mutated DNA. Data points represent the fluorescence signals: green dots—HEX+ signals, orange dots—FAM+/HEX+ signals, blue dots—FAM+ signals, grey dots—FAM-/HEX- signals.

For each specific assay, 20 µL of the reaction mixture was partitioned into droplets using the QX Dx Automated Droplet Generator (Bio-Rad) and subjected to PCR amplification using a thermal gradient to determine annealing extension temperature following the protocol: activation at 95 °C for 10 min, followed by 40 cycles of denaturation at 95 °C for 30 s and annealing range from 55.0 °C to 60 °C for 1 min using a slow ramping rate of 2 °C per second between denaturation to annealing. A final enzyme inactivation step was performed at 98 °C for 10 min, followed by a holding step at 4 °C. To optimize the laboratory routine activity, a common annealing temperature was fixed at 56.5 °C for all the assays as the optimal one for separation in 4 clusters (Figure 3). Post-PCR analysis was conducted using QuantaSoft software version 1.7.4 (Bio-Rad). Wells containing at least 20,000 droplets per well were accepted to ensure assay performance and enable data comparability with other laboratories.

### 3.4. Validation Assay

Positivity thresholds were manually defined for each sample, as signal-to-background ratios varied depending on the specific codon and mutation type. The quantity of loaded DNA was also found to affect false-positive droplet formation. Therefore, to preserve assay sensitivity during follow-up, approximately 80 ng of sample was split into four reactions. A minimum of three FAM-positive droplets was required to define a positive mutation call, in accordance with standard practice and manufacturer recommendations.

The probability distribution of false-positive events in negative controls (LOB95%) was evaluated by measuring 29 representative blank samples for *IDH1* R132H, 30 for *IDH1* R132C, *IDH*2 R140Q, and 40 for *IDH*2 R172K assays. To establish the LOD95%, we evaluated the statistical distributions of both true-positive and false-positive results. Four mutated patient DNA samples, representative of each mutation under study, were serially diluted in wild-type (WT) DNA to generate standards with VAFs ranging from 48.7% to 0.05% for *IDH1* R132H, from 35.2% to 0.05% for *IDH*2 R172K, from 48.6% to 0.05% for *IDH1* R140Q and from 8.15% to 0.05% for *IDH1* R132C. Linearity, sensitivity and intra-run reproducibility of the assays were assessed by analyzing eight replicates at each dilution point. LOB and LOD values were determined using a droplet count-based approach, following the CLSI EP17-A2 guideline [23]. The LOB was calculated as the 95th percentile (non-parametric) of the observed distribution of mutant droplet counts. This value was used as the threshold for determining the LOD which was established empirically: for each dilution point, the number of replicates in which the mutant droplet count exceeded the LOB was determined. The LOD was defined as the highest dilution point (i.e., the lowest concentration tested) at which at least 95% of replicates showed a number of mutant droplets above the LOB. Additionally, the LOB was calculated in terms of target-specific concentration (LOB, in copies/µL), based on the output from the software using only droplets positive for the target channel. This value was reported for descriptive purposes only and was not used in LOD determination.

### 3.5. Assessing the Relationship Between IDH and NPM1 Mutation Dynamics

To assess the longitudinal relationship between *IDH* mutation burden and *NPM1* mutation burden in patients harboring both mutations, we employed Generalized Estimating Equations (GEE), a population-averaged modeling approach suitable for repeated measures data. Fractional abundance of *IDH* mutations (measured by ddPCR assays) and relative quantification (RQ Ratio) of *NPM*1 mutations (measured by qPCR) were analyzed as continuous variables across serial timepoints. The samples with both *NPM*1 and *IDH1-2* values below the detection limit were excluded from correlation analyses. This choice reflects the aim of evaluating whether *IDH1* fractional abundance reliably mirrors *NPM*1 dynamics in samples where at least one marker is detectable, as double-undetectable cases do not contribute information on marker co-variation.

GEE models were fitted using an identity link function and Gaussian family, with patient ID specified as the clustering variable to account for intra-individual correlation. Given the irregular temporal structure and non-decaying within-subject correlation observed in the data, we selected a working correlation structure of type “unstructured”, which imposes no assumptions on the pattern of correlation among repeated measures. To assess the robustness of the observed association, we also performed a rank-based GEE analysis, transforming both variables into ranks prior to modeling. This approach mimics the logic of a Spearman correlation while appropriately adjusting for repeated measurements. All models were estimated using the geeglm (.) function from the geepack package in R version 4.4.3 (28 February 2025) on RStudio v. 2024.12.1. Statistical significance was defined as a two-sided *p*-value < 0.05.

## 4. Conclusions

The prognostic significance of *IDH* mutations in Acute Myeloid Leukemia remains a subject of ongoing debate, influenced by the clinical context, co-occurring genetic alterations, and specific mutational hotspots [24]. While qPCR is the established gold standard for monitoring well-known markers like *NPM1, CBFB-MYH11*, and *RUNX1:RUNX1T1* [2], *IDH* mutation monitoring shows promise for measurable residual disease tracking, particularly in patients who lack other detectable molecular alterations and for whom commercial kits are unavailable [15].

Ongoing advancements in molecular technologies offer increasingly powerful methods for detecting a broad spectrum of targetable biomarkers. This necessitates the adoption of new, sensitive approaches to quantitatively evaluate treatment effectiveness by measuring the reduction or persistence of mutated clones. The European LeukemiaNet International Working Group for MRD Assessment and Validation in AML (ELN-DAVID) expert panel, as published by Ravandi et al., recommends utilizing next-generation sequencing, PCR (qPCR, ddPCR), and multiplexed immunophenotyping for MRD detection and monitoring [25]. Given the lengthy turnaround time of NGS and its inherent error rate (typically 0.1% to 1%), which often limits its practical sensitivity for MRD, ddPCR can be a valuable complementary and sometimes preferred method for monitoring mutation burden at very low levels. However, a significant obstacle is the lack of standardized, validated commercial ddPCR assays and the absence of guidelines for PCR settings. This requires each laboratory to establish its own in-house routine conditions and cutoff values for each specific target, potentially leading to variability in results between laboratories and impacting the clinical interpretation of MRD levels. For clinical applications, new ddPCR tests need standardized validation to ensure reliable and consistent MRD measurement.

In a study of 215 AML patients, *IDH* mutations were observed in 45 patients (20.9%). Specifically, 10 patients carried a single nucleotide variant on codon 132 (R132H—12.77% and R132C—8.51%), 28 on codon 140 (R140—59.57%), and 7 on codon 172 (14.89%). It was noted that *IDH1* and *IDH*2 mutations were mutually exclusive within this cohort.

This study focused on four molecular ddPCR-specific assays designed to identify the most frequent hotspot mutations (*IDH1*-R132H, *IDH1*-R132C, *IDH*2-R140Q, and *IDH*2-R172K) found in the AML patient cohort. The objective was to establish and optimize these ddPCR-specific assays to assess their technical feasibility for quantitative MRD monitoring within clinical units.

The optimized assays developed in this work demonstrated high sensitivity for detecting and quantifying hotspot mutations. The LOD was estimated at 0.07% for *IDH1* R132H, 0.2% for *IDH1* R132C, and 0.1% for *IDH1* R140Q and R172K codons.

To indirectly validate the role of quantitative *IDH* mutation analysis by ddPCR as an MRD marker in AML, six patients co-harboring both *IDH* and *NPM1* mutations were studied. GEE analysis revealed that *NPM1* and *IDH* mutations exhibited largely parallel patterns. These findings suggest that *IDH* mutations could serve as a valuable surrogate marker for disease monitoring, similar to *NPM1*, especially in patients lacking other quantifiable MRD markers.

Unfortunately, a comparative analysis with established MRD detection methods in patients lacking molecular alterations could not be conducted. Therefore, despite the encouraging findings, the clinical utility of *IDH* mutation monitoring by ddPCR in the follow-up of AML patients remains to be fully defined.

## Figures and Tables

**Table 1:** DNA samples originating from AML patients with mutations in *IDH1* or *IDH*2 used for ddPCR quantification.

Gene	Variant	Number of Patients	Percentage of Total IDH-Mutated Patients
*IDH1*	R132C	4	8.51%
*IDH1*	R132H	6	12.77%
*IDH2*	R140Q	28	59.57%
*IDH2*	R172K	7	14.89%
Total IDH-mutated	45 (10 IDH1 + 35 IDH2)		100%
Total DNA samples analyzed:	205		
At diagnosis	45		
During Follow-up	160		

**Table 2:** Limit of Blank Data.

Mutation	Fractional Abundance (FA)	Blank Replicates (n)	Average Mutant Copies/µL	Average Wild-Type Molecules/µL	Positive Droplets (FAM+/HEX-)	Average Droplets
*IDH1* R132H	0.05%	29	0.5	917,600	0	20,157.00
*IDH2* R140Q	0.02%	30	0.2	774,480	0	21,638.00
*IDH1* R132C	0.04%	40	0.2	485,000	0	20,904.00
*IDH2* R172K	0.04%	30	0.3	805,870	2	21,660.00

**Table 3:** Limit of Detection value data. Assessment for *IDH*1 and *IDH*2 mutations via serial dilution ddPCR.

Mutation	Dilution Point (LL Samples)	Replicates Exceeding LoB	Theoretical Mutant Copies/µL (LoD)	Fractional Abundance (FA) at LoD	Merged Mutation Droplet Count	Merged WT Droplet Count
*IDH1* R132H	7	8/8	0.6	0.07%	93	174,268
*IDH2* R140Q	7	8/8	1	0.1%	158	181,644
*IDH1* R132C	6	8/8	0.4	0.2%	53	171,795
*IDH2* R172K	6	6/6	0.6	0.1%	53	110,277

**Table 4:** Sample set of patients used in the analysis of IDH and NPM1 mutation dynamics.

NPM1	IDH	Number of Patients	Total Samples
Pos	*IDH2* R172K	1	12
Pos	*IDH2* R140Q	3	36
Pos	*IDH1* R132H	2	20

## Data Availability

The original contributions presented in this study are included in the article. Further inquiries can be directed to the corresponding authors.

## Abbreviations

The following abbreviations are used in this manuscript:

(D)-2-HG	(D)-2-Hydroxyglutarate
α-KG	α-ketoglutarate
AML	acute myeloid leukemia
DNA	deoxyribonucleic acid
ddPCR	droplet digital polymerase chain reaction
IDH	isocitrate dehydrogenase
LOB	limit of blank
LOD	limit of detection
MDS	myelodysplastic syndromes
NGS	next-generation sequencing
qPCR	quantitative polymerase chain reaction
VAF	variant allele frequency
FA	fractional abundance
MRD	measurable residual disease
ELN	The European LeukemiaNet
RQ Ratio	Relative Quantification Ratio
